# The mediating effects of quality of life, depression, and generalized anxiety on perceived barriers to employment success for people diagnosed with Neurofibromatosis Type 1

**DOI:** 10.1186/s13023-021-01866-6

**Published:** 2021-05-21

**Authors:** Frank D. Buono, Matthew E. Sprong, Erina Paul, Staci Martin, Kaitlyn Larkin, Amir Garakani

**Affiliations:** 1 Department of Psychiatry, Yale School of Medicine, 300 George Street, New Haven, CT 06510 USA; 2 Lock Haven University, Lock Haven, PA USA; 3 Merck & Co., Inc, Rahway, NJ USA; 4 Pediatric Oncology Branch, Center for Cancer Research, National Cancer Institute, NIH, Bethesda, MD 20892 USA; 5 Icahn School of Medicine At Mount Sinai, New York, NY USA

**Keywords:** Neurofibromatosis Type 1, Quality of life, Mediation analysis, Depression, Barriers to successful employment

## Abstract

**Background:**

Neurofibromatosis Type 1 (NF1) is a genetic disorder that presents with physical symptoms that can negatively impact numerous areas of one’s life, including occupational and psychological functioning, with decreased quality of life compared to a normative population. The purpose of the current study was to explore differences in the impact of psychological factors (anxiety and depression), quality of life and employment hope on barriers to successful employment between those with NF1 and matched controls.

**Methods:**

A total of 212 individuals were stratified into two groups (NF1 and matched controls) using a cross-sectional design that collected a one-time response.

**Results:**

A mediation analysis in which total barriers to successful employment on the differences between groups with quality of life, anxiety and depression as the mediators, and levels of employment hope as the co-variates were examined. The results confirmed a direct (.001) and indirect (< .001) relationship between barriers to successful employment with NF1 to matched controls, and with quality of life, anxiety, and depression.

**Conclusions:**

The current findings indicate that the barriers to successful employment for individuals with NF1 impact their quality of life, anxiety, and depression more than that of the matched controls. Poorer barriers of employment observed amongst people with a genetic disease can impact mental health and quality of life.

## Introduction

Neurofibromatosis Type 1 (NF1) is an autosomal dominant genetic disorder affecting an estimated 1 in 3000 individuals worldwide [1]. Typical presentations of NF1 can include plexiform neurofibroma tumors (PNs) (large tumors that grow from nerves and can distort the face, limbs, back, chest or abdomen), café au lait macules (dark pigmented spots on skin) [2, 3], and extensive dermal neurofibromas, which are tumors that present as bumps or lumps on the skin [4]. These highly visible and cosmetically disfiguring features of NF1 can leave individuals vulnerable to stigmatization and other social emotional difficulties such as depression, anxiety, and low self-esteem [5]. The direct appearance effects of NF1 physical symptoms and the subsequent social emotional consequences can negatively impact numerous areas of one’s life, including occupational functioning [6, 7].

Individuals with NF1 cope with the unpredictable nature of the disease that varies in severity and medical complexity [8] and therefore at increased risk for depression and anxiety [9]. A recent survey of 498 individuals with NF1 conducted by Cohen et al. [5] found that 55% of all participants indicated a high likelihood for clinical depression. In addition, Pasini et al. [10] reported that children and young adults with NF1 had higher predisposition of developing an anxiety disorder. Even without a genetic disorder, depression or anxiety can have a direct negative impact on an individual’s Quality of Life (QoL) [6].

QoL is a multidimensional construct that represents the amalgamation of factors that affect a person’s life including emotional, physical and social influences [11, 12]. It has been documented that individuals with NF1 experience decreased QoL compared to the general population [13]. In addition, employment status has been shown to be a strong predictor of QoL, with employed individuals reporting greater QoL than those who are unemployed [14, 15]. Previous research has shown that perceived QoL is negatively impacted when individuals are not employed [16]. Likewise, research has shown that prolonged job insecurity (subjective perception or feelings of insecurity about the future of one’s employment) has been negatively associated with psychological well-being [17]. Furthermore, Krueger et al. [18] found that the longer an individual is unemployed, the higher the dissatisfaction and level of unhappiness with their lives.

Although research has been conducted on QoL within NF1 populations regarding physical appearance, anxiety and depression [19], little research has examined how disease-related barriers to successful employment impact the QoL, anxiety or depression in individuals with NF1. The purpose of the current study was to explore differences in the impact of psychological factors (anxiety and depression), QoL and employment hope on barriers to successful employment between those with NF1 and matched controls. We hypothesized that individuals with NF1 would have increased barriers to successful employment and employment hope, while having decreased QoL as compared to matched controls. Additionally, we hypothesized that individuals with NF1 will suffer increased generalized anxiety and depressive symptoms due to the perceived barriers to successful employment compared with controls.

## Methods

### Participants

Two groups were recruited between March 2020 and August 2020: Individuals with NF1 and Matched Controls. Both groups were convenience samples, recruited by different means. For the NF1 group, after acquiring approval from both executive directors across two NF advocacy listservs (NF Network and NF Northeast) an initial email was sent out to 200 registered individuals, of which 128 adults responded. Of the respondents, 105 completed the assessments and met criterion (did not fully complete surveys n = 12; different diagnosis n = 8; under 18 n = 3). Matched Controls were recruited via Qualtrics recruitment services, of which 115 individuals were initially recruited. Of the respondents, 108 successfully completed the assessments (did not fully complete surveys n = 7). The inclusion criterion for the study were as follows: 1) are at least 18 years old, 2) have a current documented case of NF1; 2a) no documented case of NF1 (matched controls). The exclusion criteria were the following: 1) unable to read or understand English at a 5th grade level, 2) no access to internet via computer, tablet, or smart phone, 3) diagnosis of NF2, and/or Schwannomatosis, 3a) has been diagnosed and is living with a current medical serious condition (matched controls).

### Assessments

The Generalized Anxiety Disorder Scale-7 (GAD-7) is a 7-item, self-rated scale developed in correspondence to the Diagnostic and Statistical Manual and updated for the 5th Edition (DSM-5), and is used as a screening tool and severity indicator for GAD [20]. Each statement, one of four choices are provided, and respondents can select one response (*1* = *not at all, 2* = *several days, 3* = *over half days, 4* = *nearly every day*). Each column is then added, and a total score is obtained, with scores falling into four levels of anxiety, including minimal (1–4), mild (5–9), moderate (10–14), and severe (15–21). Reliability of this assessment demonstrated a Cronbach’s alpha of 0.92 (α = 0.92) [20].

The Patient Health Questionnaire (PHQ-9) is a nine-item, self-report scale that is used for screening, diagnosing, monitoring, and measuring the severity of depression (mild [scores of 5–9], moderate [scores of 10–14], moderately severe [scores of 15–19], severe [scores of 20–2]) [21]. Respondents indicate how bothered they were from each problem over the past 2 weeks by selecting one of four choices (*1* = *not at all, 2* = *several days, 3* = *over half days, 4* = *nearly every day*). Each column is then added, and a total score is obtained. Reliability of this assessment demonstrated a Cronbach’s alpha of 0.89 (α = 0.89) [21].

The Employment Hope Survey-Short (EHS-14) is a 14-item, self-report Likert scale based on the 24-item six-factor EHS. The Short-EHS maintains the 0 (strongly disagree)-10 (strongly agree) rating; however, it evaluates four factors (psychological empowerment, futuristic self-motivation, utilization of skills and resources, and goal orientation). Overall coefficient alpha and those for the factors of the Psychological Empowerment, Futuristic Self-Motivation, Utilization of Skills and Resources, and Goal Orientation subscales were 0.932, 0.949, 0.833, 0.949, and 0.931, respectively [22].

The Barriers to Employment Success Inventory (BESI), Fifth Edition, is 50-item scale designed to assist an individual identifying and exploring the types of potential barriers that are keeping them from obtaining a good job, enjoying employment success, and advancing in their career [23]. Participants indicate one of four choices (*1* = *no concern, 4* = *greatest concern*) that assess five barrier categories, including Personal and Financial, Emotional and Physical, Career Decision-Making and Planning, Job-Seeking Knowledge, and Education and Training. Sub-scores are cumulated and reported as an aggregate value. Reliability of the BESI sub-factors have demonstrated a Cronbach’s alphas ranging between 0.87 and 0.95 [23].

The Short-form Health Survey for the Medical Outcomes Study (SF-12) is a health-related quality-of-life scale that measures dimensions both for functioning (physical, social and role) and for well-being (mental health, health perception and pain). It yields six numerical scores that are aggregated into a (0–100) parameter, where a higher score indicates better functioning or well-being [24].

### Procedure

The current study was approved by the first author university’s Human Subjects Committee and abides by the Helsinki Code of Ethics (1975, 2000). The current study was a cross-sectional design quantitative method that collected a one-time response from participants. Participants matched across both groups: gender (at minimum 45% were female), and age (18 and over). All data collected from questionnaires were anonymized by the first author (FB) and stored in a secure online server hosted by the first author’s institution.

Individuals who agreed to participate in the study were instructed to click on the link at the bottom of the recruitment email. The link relocated the study participants to a password protected survey within *Qualtrics*. Prior to completing the assessments, potential participants were welcomed to the survey and requested to read and electronically sign (e-sign) the informed consent form that described the study, how the data would be used, and how it was recorded. Copies of the informed consents were securely emailed to participants. Participants were stratified into two groups, based on answering research questions (“Do you have Neurofibromatosis?” and “If marked yes, what type of Neurofibromatosis do you have”): NF1 and Matched Controls. Once participants completed the assessments, a debriefing statement was provided. Four $50 USD Amazon gift cards were randomly drawn and distributed based on the completion of the assessments.

### Data analysis

Analyses were conducted using R version 3.6.3. Descriptive statistics were calculated to summarize demographic and disease-related information and were compared in one-way ANOVAs or independent t-tests. The level of statistical significance was set at p < 0.05 for all computations. For the mediation analysis, the indirect, direct, and total effects of the total barriers to employment success (Barriers) on the differences between groups (Group) with quality of life (M1), anxiety (M2) and depression (M3) as the mediators, and employment hope sub-factors as the co-variates were examined to illustrate the use of methods of mediation analysis. The goal of this model was to investigate the total (Barriers) and direct effects (Group). It also investigated the indirect effect (IE) obtained from the product of coefficients. These influence measures were treated as both continuous and/or binary with the latter formed by dichotomization of the naturally continuous predictors in R syntax. Bootstrapping was applied to empirically estimate the sampling distribution of the indirect effect and generate a bootstrap confidence interval (95% CI) based on 10,000 bootstrap samples for bias corrected bootstrap CIs. Standard errors (SE) and confidence intervals (CI) were obtained, in the mediation analysis as recommended in Valeri & Vanderweele [25].

## Results

### Descriptive characteristics

In the NF1 Group, the average age was 43.6 years (SD = 12.5; Range 18–56) with 66% (n = 69) being female, as compared to the Matched Controls whose average age was 30.1 years (SD = 10.2; Range 18–52), and 56% (n = 56) were female. A majority of each group (NF1: 65% [N = 70]; Matched Control: 62% [N = 67]) were actively employed, with only a small minority who were not employed (NF1: 11% [N = 12]; Matched Controls: 16% [N = 17]). In all cases of health-related issues, significant differences were noted between NF1 and Matched Controls. The remaining participant demographics and characteristics are displayed in Table 1. Significant differences between generalized anxiety, depression, QoL, barriers to successful employment and employment hope across groups are shown in Table 2.

**Table 1:** Demographics and Medical Information across Groups

	NF group (N = 105)	Matched control (N = 108)	p value
Age	43.6 (12.5)	30.1 (10.2)	
Gender identification; N (%)			0.38
Male	35 (33%)	45 (42%)	
Female	69 (66%)	60 (56%)	
Transgender	0	2 (2%)	
Other	1 (1%)	2 (2%)	
Marital status; N (%)			0.28
Single	46 (43%)	55 (50%)	
Married	43 (41%)	42 (39%)	
Divorced	9 (9%)	6 (6%)	
Separated	2 (2%)	2 (2%)	
Widowed	3 (3%)	0	
Other	2 (2%)	3 (3%)	
Highest education			0.009
High school/GED	21 (20%)	37 (34%)	
Technical degree	13 (12%)	7 (6%)	
Some of college	9 (9%)	8 (7%)	
Bachelor’s degree	12 (11%)	13 (12%)	
Advanced degree	12 (11%)	7 (6%)	
Work status			.84
Employed	70 (65%)	67 (62%)	
Student	5 (5%)	13 (12%)	
Retired	5 (5%)	3 (3%)	
Worker's compensation	1 (1%)	1 (1%)	
Receive SSD/SSI	12 (11%)	6 (6%)	
Unemployed	12 (11%)	17 (16%)	
Health related illnesses; yes Responses (%)			
Respiratory/breathing/pulmonary	30 (29%)	14 (13%)	0.005
Cardiac	20 (19%)	11 (10%)	0.067
Gastrointestinal	49 (47%)	13 (12%)	< .001
Urinary/genital	29 (28%)	10 (9%)	< .001
Musculoskeletal	41 (40%)	13 (12%)	< .001
Rheumatologic/immunologic	26 (25%)	8 (7%)	< .001
Neurological	61 (58%)	12 (11%)	0.004

**Table 2:** Results of anxiety, depression, QoL, total barriers of employment and employment hope between groups (NF1 vs. matched control), and covariates

	NF1		MC		*t*	*df*	*p*
	*M*	SD	*M*	SD			
Generalized anxiety	*14.4*	5.91	*11.37*	4.73	4.14	211	< .001
Quality of life	*32.1*	2.80	*29.00*	6.50	4.37	211	< .001
Depression	*18.7*	6.87	*13.18*	4.98	6.74	211	< .001
Total barriers to employment	*24.1*	5.40	*19.60*	7.20	5.02	211	< .001
Employment hope—psychological flexibility	*29.6*	9.56	*26.22*	10.01	2.52	211	0.013
Employment hope—self-motivation	*12.47*	6.40	*12.48*	5.10	− 0.19	211	NS
Employment hope—goal orientation	*17.24*	9.10	*18.80*	7.50	− 1.34	211	NS
Employment hope—utilization of skills	*29.6*	9.56	*26.22*	10.01	2.52	211	0.013

	Anxiety			Depression			Quality of life		
	*ab*	SD	CI	*ab*	SD	CI	*ab*	SD	CI
CV1*	0.01	0.07	− 0.14 to 0.17	*0.11*	0.14	− 0.12 to 0.46	− *0.22*	0.14	− 0.53 to 0.01
CV2*	*0*	0	− 0.25 to 0.23	− *0.10*	0.19	− 0.54 to 0.25	− *0.15*	0.16	− 0.50 to 0.17
CV3*	− *0.09*	0.15	− 0.46 to 0.15	− *0.28*	0.25	− 0.87 to 0.09	*0.28*	0.18	− 0.04 to 0.67
CV4*	*0.02*	0.16	− 0.31 to 0.39	*0.02*	0.27	− 0.50 to 0.60	*0.36*	0.21	0.02 to 0.83

M = Mean; SD = Standard Deviation; NF1 = Neurofibromatosis tyte 1; MC = Matched Controls; df = Degrees of Freedom, NS = not significant, CI = Confidence Interval. *p < .0001

### Mediation analysis

**Fig. 1:**
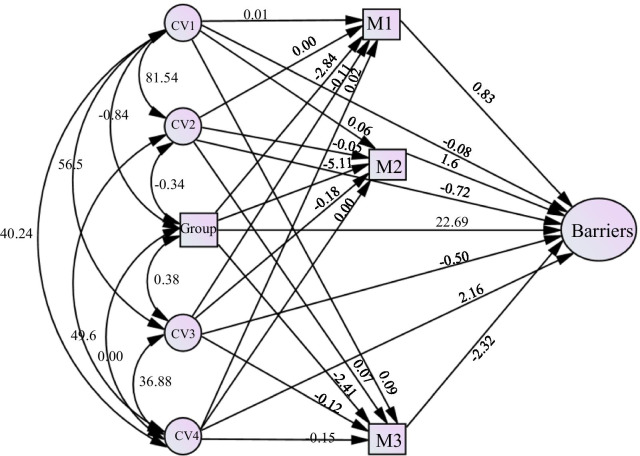
Mediation analysis of work barriers across NF1 and matched controls. Barriers = Total Score of Barriers to employment success inventory; Group = Grouping variable (NF1 or Matched Controls); M1 = Generalized Anxiety Total Score; M2 = Patient Health Questionnaire Total Score; M3 = Short Form-12 Total Score; CV1 = Employment Hope psychological empowerment subfactor; CV2 = Employment Hope futuristic self-motivation subfactor; CV3 = Employment Hope goal orientation subfactor: CV4 = Employment Hope utilization of skills and resources subfactor

A mediation analysis was performed to examine the indirect effects of generalized anxiety, depression and quality of life on the direct relationship of predictor variable (group) and outcome variable (barriers to successful employment). The levels of the Employment Hope Scale (EHS) as covariates used for this study included psychological empowerment (CV1), futuristic self-motivation (CV2), goal orientation (CV3), and utilization of skills and resources (CV4). The total effect (c) of group on total score was 17.73 (SE = 5.35, t = 3.31, df = 207, p = 0.001). The direct effect (c′) of group on total score removing mediator effects is 22.69 (SE = 5.23, t = 4.33, df = 204, p < 0.001), the indirect effect (ab) of group on total score through mediator effects was − 4.96. Also, the mean bootstrapped indirect effect was − 4.83 (SD = 4.06, CI =  − 12.72—3.24, p < 0.001). The total effect of CV1 on the total score was − 0.19 (SE = 0.42, *t* = -0.046, df = 207, p = 0.65), total effect of CV2 on total score was − 0.96 (SE = 0.58, *t* =  − 1.65, df = 207, p = 0.10), total effect of CV3 on total score was − 0.60 (SE = 0.63, *t* =  − 0.96, df = 207, p = 0.34), total effect of CV4 on total score was 2.53 (SE = 0.79, *t* = 3.20, df = 207, p = 0.0016). The direct effects for each level of the EHS includes psychological empowerment (CV1) − 0.08 (SE = 0.37, *t* =  − 0.23, df = 204, p = 0.82), futuristic self-motivation − 0.72 (SE = 0.50, *t* =  − 1.43, df = 204, p = 0.15), goal orientation − 0.50 (SE = 0.55, *t* =  − 0.91, df = 204, p = 0.36), and utilization of skills and resources 2.16 (SE = 0.69, *t* = 3.14, df = 204, p = 0.00) (Fig. 1).

### Generalized anxiety (M1), depression (M2), and quality of life (M3)

The direct effect of Anxiety was 0.83 (SE = 0.70, *t* = 1.19, df = 204, p = 0.24). The direct effect of Depression was 1.60 (SE = 0.65, *t* = 2.47, df = 204, p = 0.014). The direct effects of quality of life was − 2.32 (SE = 0.44, *t* =  − 5.21, df = 204, p ≤ 0.001). The covariates used within the mediation analysis include psychological empowerment, futuristic self-motivation, goal orientation, and utilization of skills and resources. The indirect effects (ab) of CV1, CV2, CV3, and CV4 are presented in Table 2, and were all significant (p < 0.0001).

## Discussion

The current manuscript investigated the impact of depression, generalized anxiety, quality of life, and employment hope on barriers to successful employment within individuals with NF1 compared to that of matched controls. Our results demonstrated that barriers to successful employment fully mediate quality of life, anxiety, and depression across individuals with NF1 to matched controls. Generalized anxiety, depression, and quality of life had both a direct and indirect effect, indicating a totally mediated relationship between these variables. The covariate, employment hope, had a significant direct effect, of which only two sub-factors (self-motivation and utilization of skills and resources) demonstrated a significant indirect relationship. The current findings indicate that the barriers to successful employment for individuals with NF1 impact their quality of life, anxiety, and depression more than that of the matched controls, thus emphasizing the importance of understanding of the impact of NF1 on those who experience the disease. To the authors’ knowledge, the current study is the first to evaluate these constructs within the NF1 population, providing a clinical and vocational understanding that is missing within the current research.

The obtained results have several significant theoretical, future research, and clinical implications for individuals suffering from NF1. From the theoretical perspective, when barriers to successful employment (e.g. Personal and Financial, Emotional and Physical, Career Decision Making and Planning) are eliminated or reduced, improvement of quality of life, depression and/or anxiety can be maintained [26]. Maintaining a normalized sense of quality of life when suffering from a life-long disease or disability is critically important; thus, understanding the direct and indirect effects of the mediating effect of barriers to successful employment onto quality of life, depression and anxiety, supplements the existing research understanding of NF1 and how individuals experience the disease [27–29].

From a research perspective, there were significant differences in depression between groups (M = 19 NF; M = 13 MC) but generalized anxiety did not demonstrate a significant difference between groups (M = 14 NF; M = 11 MC). These differences, however, did not impact the overall direct and indirect effects of the mediation analysis. The generalized anxiety findings did have stronger direct effects than depressive symptoms. The current findings add to the existing research that individuals with NF1 who have increased depression scores have poorer quality of life [5, 30]. Multiple articles have demonstrated that individuals with NF1 suffer from generalized anxiety [8, 10]. Indications of higher levels of anxiety can contribute to lower QoL [6, 8]; however, more research is needed to evaluate how barriers to successful employment can affect generalized anxiety. A recent meta-analysis concluded that QoL affects the well-being of individuals because of its impact onto physical, emotional, and cognitive functioning [31]. Looking at aggregate values, total barriers to successful employment were significantly higher for the control group than individuals with NF1, providing that while barriers such as physical, mental, social and societal pressures are constrained on individuals with NF1, the perception of barriers to successful employment may be perceived as secondary from a QoL perspective.

The way in which employment hope impacted the direct and indirect effects of the current study is compelling. Hong et al. [22] discussed employment hope as a necessity in obtaining employment, as it increases the individual’s opportunity to achieve employment. The immediate impact of employment hope has been found to influence sustainability of work related employment [32]. Yet, the indirect effects indicate that only self-motivation and utilization of skills can explain the observed relationship. This provides some initial understanding and explanation for the current findings, since the preexisting lifetime congenital condition may lead to a higher tolerance or wherewithal in individuals with NF1.

From a clinical perspective, the current findings echo previous reports that poor mental health is the consequence of and risk factor for unemployment, and that poorer mental health can be risk factor subsequent consequence of employment [33]. Overwhelmingly, and understandably, the physical manifestations of NF1 take precedent; however, other facets of QoL measures including active employment can provide a viable escape from the previous accompaniments, along with social integration and feelings of accomplishments. Given that individuals with NF1 have greater likelihood of having dyslexia and/or learning disabilities, individuals with NF1 may potentially require more external support in the workplace [1, 34]. Assistance programs, such as vocational rehabilitation, can customize services to meet the needs of individuals diagnosed with NF1, therefore potentially increasing labor force participation and decreasing psychological, physical, vocational, and social barriers related to successful employment [16]. Further research is needed to understand the impact of barriers to successful employment for individuals with NF1. Maintaining normalcy for individuals with NF1 is an arduous task given the multiple and complex medical, psychological, emotional and occupational deficits which can accompany the disease.

The current study is not without limitations. Initially, the current study was a cross-sectional design with an under-powered sample of individuals with NF1. Although an inference or causal relationship cannot be found using cross-sectional methodology, the current study can provide future directions for researchers on the impact of anxiety and depression on barriers to successful employment. Relative to other large-scale research studies with NF1 adults [5, 35], the current study has a modest representation of individuals with NF1. Given the rarity of the disease, a well-powered study is difficult to attain. Additional research should attempt to increase the sample size, thus allowing for a greater external validity of the results. Lastly, typical QoL measures can include questions regarding employment. Yet, parsing out the barriers of successful employment and employment hope (QoL sub-factors) within the NF1 population has not been completed. Future research should investigate the underlying features of the current study (e.g., barriers to successful employment, employment hope, quality of life, depression and generalized anxiety) in a more clinical capacity through a more systematic process. By including other variables that can affect quality of life functioning will allow for clearer causation of differentiation. Additionally, the impact of barriers to successful employment across NF1, NF2 and Schwannomatosis populations should be considered, given each presentation of the corresponding diseases are inherently different.

## Conclusions

The current article is the first to demonstrate that quality of life can directly be affected by barriers of successful employment within the NF1 population. Results extend existing research by providing insight on the impact of quality of life on employment. Poorer barriers of employment observed amongst people with NF1 can impact both mental health and quality of life. Through better understanding the factors that negatively impact barriers to successful employment, assistance programs can tailor services for individuals with NF1, potentially increasing labor force participation and QoL.

## Data Availability

The data that support the findings of this study are available on request from the corresponding author. The data are not publicly available due to privacy or ethical restrictions.
